# DNA methylation dysregulations in rheumatic heart valve disease

**DOI:** 10.1186/s12872-017-0585-3

**Published:** 2017-06-17

**Authors:** Kangjun Shen, Hui Liu, Ran Jing, Jiangfeng Yi, Xinmin Zhou

**Affiliations:** 1 Department of Cardiovascular Surgery, The Second Xiangya Hospital of Central South University, 139. Renmin Road, Changsha, Hunan 410011 People’s Republic of China; 2 Department of Hemodialysis Center, The Third Xiangya Hospital of Central South University, Changsha, Hunan People’s Republic of China; 3 Department of Cardiology, The Xiangya Hospital of Central South University, Changsha, Hunan People’s Republic of China

**Keywords:** Rheumatic heart valve disease, DNA methylation, Dysregulation

## Abstract

**Background:**

The epigenetic changes underlying the development of rheumatic heart valve disease (RHVD) remain incompletely understood. Limited evidence suggests that abnormal DNA methylation might be involved in the pathogenesis of RHVD. In the present study, we evaluated the DNA methylation dysregulations from myocardial tissue in RHVD patients systematically.

**Methods:**

Right atrial myocardial tissue obtained from rheumatic valvular patients who had undergone valve replacements surgery (*n* = 73) and were compared to healthy controls (*n* = 4). the promoter methylation level of Intercellular adhesion molecule-1 (ICAM-1) gene and its correlation with ICAM-1 mRNA expression level, the global DNA methylation level and its correlation with age and mRNA expression level of DNA methyltransferase (DNMT) genes were detected.

**Results:**

The ICAM-1 mRNA expression was increased (healthy control vs. NHYA III, 0.70 ± 0.19 vs. 4.38 ± 3.19, *p* = 0.011; NYHA IIvs. NHYA III, 2.60 ± 1.99 vs. 4.38 ± 3.19, *p* = 0.008) and the ICAM-1 gene was hypomethylated in RHVD patients (healthy controls vs. NYHA II, 0.120 ± 0.011 vs. 0.076 ± 0.057, *p* = 0.039; healthy control vs. NHYA III, 0.120 ± 0.011 vs. 0.041 ± 0.022, *p* < 0.001; NYHA IIvs. NHYA III, 0.076 ± 0.057 vs. 0.041 ± 0.022, *p* < 0.001). Meanwhile, The ICAM-1 mRNA expression level has negative correlation with the mean methylation level in the promoter region of ICAM-1 gene (*r* = −0.459, *p* < 0.001). The global DNA methylation levels was significantly increased in RHVD patients than in healthy controls (healthy control vs. NHYA III, 0.77 ± 0.28 vs. 2.09 ± 1.20, *p* = 0.017; NYHA IIvs. NHYA III, 1.57 ± 0.78 vs. 2.09 ± 1.20, *p* = 0.040) and had positive correlation with age (*r* = 0.326, *p* = 0.005), especially for older age group (≥ 60 years). DNMT1 likely plays an essential role in the DNA dysregulations in RHVD patients.

**Conclusions:**

Our analysis revealed that DNA methylation dysregulations may be relevant in the pathogenesis of RHVD.

## Background

Rheumatic heart valve disease (RHVD) refers to the chronic heart valve damage which occurs after acute rheumatic fever. The progression of valve damage eventually leads to congestive heart failure [1]. The incidence and prevalence of RHVD has been decreasing significantly in developed countries. However, in most developing countries, such as China, RHVD remains the main cause of heart valve disease. It is reported that more than 15 million RHVD patients in the worldwide and 282,000 new cases are diagnosed annually [2].

The pathogenesis of RHVD is complex and still remains incompletely understood. Gene expression deregulation, inflammation and auto-immune response were all involved in the pathogenesis [3, 4]. Increasing evidences had been showed that DNA methylation dysregulation plays an important role in the occurrence and development of complex diseases in recent years. DNA methylation can lead to gene expression alternation without DNA sequence changes [5, 6]. DNA methylation regulations mainly affect the promoter regions of gene and lead to transcriptional regulation. Promoter hypomethylation correlates with increased gene expression, whereas hypermethylation relates to decreased gene expression [7, 8]. Global DNA methylation dysregulation will cause chromosomal instability [9, 10]. The role of DNA methyltransferase (DNMT) is to establish and maintain these regulations [7, 11, 12]. However, most DNA methylation deregulation studies have focused on cancer and auto-immune disease [13, 14]. The impact of DNA methylation upon the initiation/progression of cardiovascular diseases has not been investigated in detail [15].

In the present study, we detected the DNA methylation dysregulations in human atrial tissue between RHVD patients and healthy controls. The abnormal methylation status of Intercellular adhesion molecule-1 (ICAM-1) gene, the global DNA methylation and DNA methyltransferase (DNMT) may present a potential therapy strategy for RHVD patients.

## Methods

### Human right atrial myocardium

Right atrial myocardium tissue was collected from 73 RHVD patients undergoing valve replacement (All patients had no other cardiovascular diseases, such as hypertension, coronary heart disease, etc.). The average age of these RHVD patients is 46.9 ± 9.85 years; Among these 73 patients, 29 were New York heart association (NYHA) classification II and 44 were NYHA classification III (Using the NYHA functional classification [16, 17]). The patient details were listed in Table 1. We also have 4 healthy controls (Male, 44.3 ± 6.70 years old). The 4 healthy controls were heart transplant donors and are therefore deceased. The whole hearts were removed after preservation and transported in cold cardioplegia solution [18]. Right atrial tissues were cut and frozen immediately in liquid nitrogen for DNA and RNA isolation.

**Table 1:** Individual details of healthy controls and 73 RHVD patients

ITEMS	Healthy controls	NYHA II	NYHA III	P
Male: Female (cases)	4 : 0	8 : 21	19 : 25	N/A
Age (years)	44.3 ± 6.70	45.8 ± 10.0	47.7 ± 9.77	> 0.05
Left atrial dimensions (mm)	N/A	53.7 ± 11.2	58.4 ± 13.2	> 0.05
Mitral Valve involvement (N/%)	N/A	29 (100%)	44 (100%)	N/A
Aortic valve involvement (N/%)	N/A	9 (31.0%)	20 (45.5%)	N/A
Stenosis grade	N/A	2.10 ± 0.72	2.52 ± 0.66	0.015*

Stenosis grade: 1. Mild; 2. Moderate; 3. Severe

* *P*<0.05

### RNA isolation and real-time quantitative reverse transcriptase-polymerase chain reaction (RT-PCR)

Total RNA was isolated according to the Invitrogen’s standard Trizol protocol. RT-PCR was performed and the quantification of mRNA levels was adjusted by β-actin. The primers of ICAM-1, DNMT1, DNMT3a, DNMT3b and β-actin were listed in Table 2.

**Table 2:** Primer sequences used for Real-Time Quantitative RT-PCR

Gene	Primer Sequence
ICAM-1/F	CCCCGTTGCCTAAAAAGG
ICAM-1/R	TTGCACATTGCTCAGTTCATACAC
DNMT1/F	AAGAACGGCATCCTGTACCGAGTT
DNMT1/R	TGCTGCCTTTGATGTAGTCGGAGT
DNMT3a/F	TTTGAGTTCTACCGCCTCCTGCAT
DNMT3a/R	GTGCAGCTGACACTTCTTTGGCAT
DNMT3b/F	AGTGTGTGAGGAGTCCATTGCTGT
DNMT3b/R	GCTTCCGCCAATCACCAAGTCAAA
β-actin/F	GCACCACACCTTCTACAATGAGC
β-actin/R	GGATAGCACAGCCTGGATAGCAAC

### Genomic DNA extraction and DNA methylation sequencing

DNA was isolated according to the Qiagen’s protocol used by Long et al. [19]. Briefly, DNA was treated with the EpiTect bisulfate kit after isolation. The gene promoter sequences were amplified and cloned. For each samples, 10 independent clones were sequenced [20]. The primers of ICAM-1 gene were listed in Table 3.

**Table 3:** Primer sequences used for Bisulfite genomic sequencing polymerase chain reaction assay

Gene	Primer Sequence
ICAM-1/F	TTAGGGGGAGGGGTATTTTTTAGTG
ICAM-1/R	TCGCTAACCGCTTCAACTCCGAAAT

### Measurement of global DNA methylation by an ELISA-like reaction

Global DNA methylation was detected according to the Epigentek’s protocol used by Chen et al. [12]. The methylated global DNA sequences were recognized and quantified use ELISA reaction. The expression levels of global DNA methylation were adjusted to the optical density (OD) and thus can be calculated and compared.

### Statistical analysis

The one-way ANOVA analysis was used to test for differences among the three groups. Pearson’s correlation coefficient was used for the correlations analysis between the DNA methylation changes and the clinical characteristics. *P* < 0.05 was considered significant. The SPSS 16.0 software was used to carry out statistical analyses. Data was presented as mean ± standard deviation (SD).

## Results

### The mRNA expression level and the promoter methylation pattern of ICAM-1 gene

**Fig. 1:**
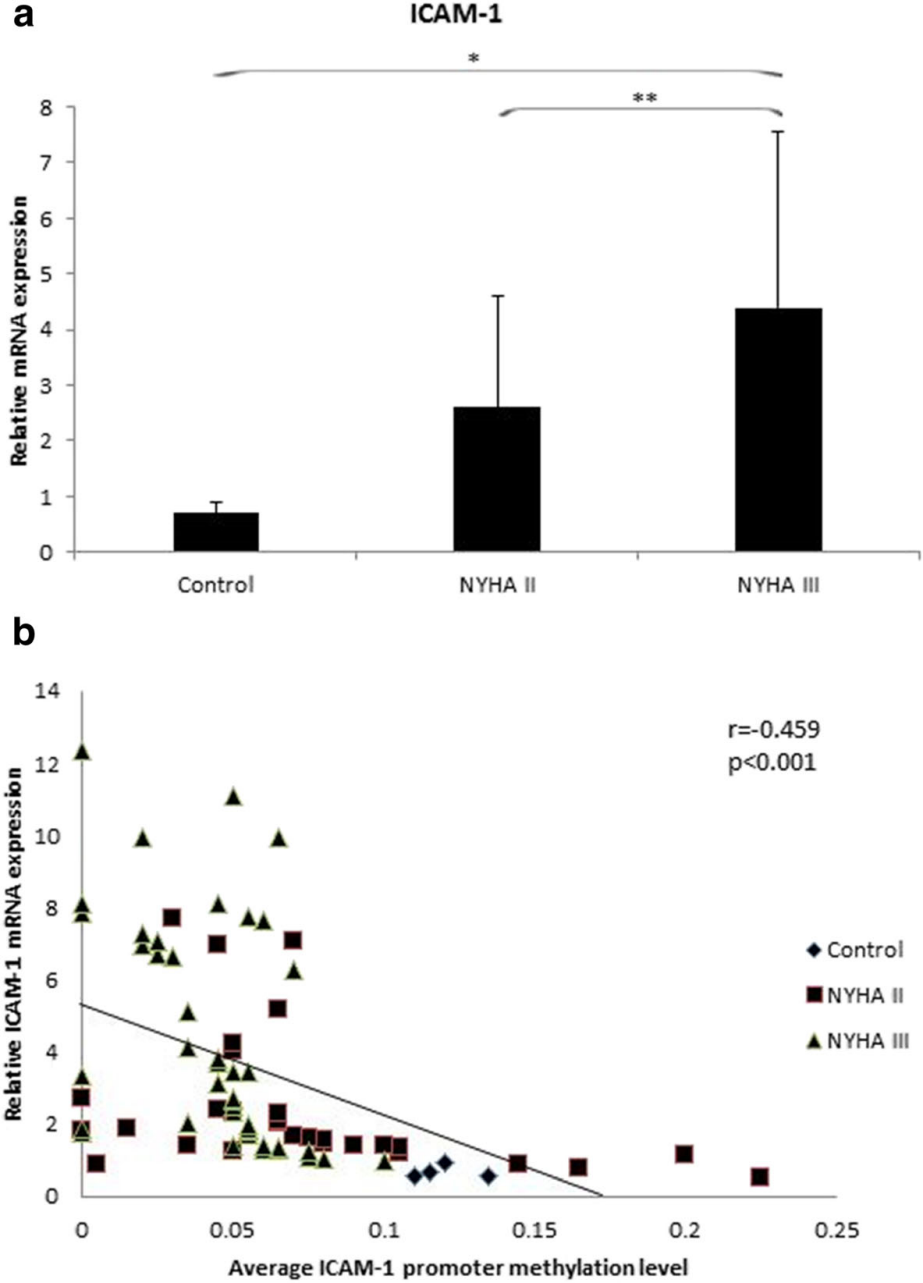
**a** Shows the mRNA expression of ICAM-1 gene in right atrial myocardium samples. The expression levels of ICAM-1 mRNA was significantly increased in NYHA III group, compared with healthy control and NYHA II group. (^*^, *p* < 0.05; ^**^, *p* < 0.01). **b** Shows the expression levels of ICAM-1 mRNA had negative correlation with the mean methylation status in the promoter region (*r* = −0.459, *p* < 0.001)

Compared with healthy controls and NYHA II patients, the mRNA levels were significantly increased in NHYA III patients (healthy control vs. NHYA III, 0.70 ± 0.19 vs. 4.38 ± 3.19, *p* = 0.011; NYHA IIvs. NHYA III, 2.60 ± 1.99 vs. 4.38 ± 3.19, *p* = 0.008). No significant difference was found between healthy controls and NYHA II patients (*P* = 0.194) (Fig. 1a).

**Fig. 2:**
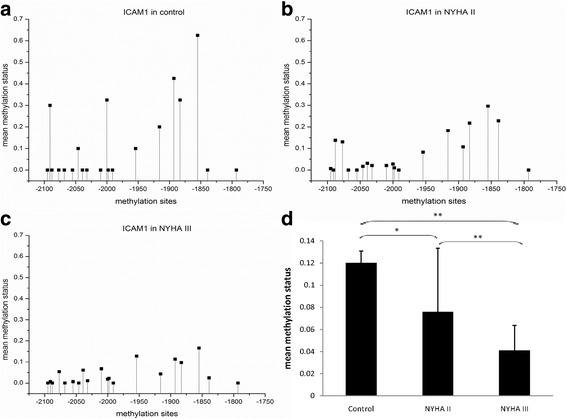
**a**, **b** and **c** Show the mean methylation status of each CG pairs in the promoter region of ICAM-1 gene in healthy control, NYHA IIgroup and NYHA III group respectively). Compared with healthy control, the promoter region methylation status with 20 CG pairs (−2095, −2091, −2088, −2077, −2068, −2055,-2046, −2039, −2032, −2010, −2000, −1998, −1991, −1954, −1916, −1893, −1883, −1855, −1839 and -1793 bp) of ICAM-1 gene were gradually decreased in NYHA II and NYHA III group. **d**, the average methylation level of CpG pairs in the region (−2138 to -1759 bp) of ICAM-1 promoter is gradually decreased in NYHA IIand NYHA III group compared to healthy control. (^*^, *p* < 0.05; ^**^, *p* < 0.01)

Then we measured the methylation levels of CG pairs in 380 bp of ICAM-1 gene (position −2138 to −1759, 20 CG pairs, CpG-density 39%). The mean methylation levels were significantly decreased with the progression of RHVD (healthy controls vs. NYHA II, 0.120 ± 0.011 vs. 0.076 ± 0.057, *p* = 0.039; healthy control vs. NHYA III, 0.120 ± 0.011 vs. 0.041 ± 0.022, *p* < 0.001; NYHA IIvs. NHYA III, 0.076 ± 0.057 vs. 0.041 ± 0.022, *p* < 0.001) (Fig. 2).

The mRNA expression level of the ICAM-1 gene had negative correlation with the mean ICAM-1 promoter methylation level (*r* = −0.459, *p* < 0.001) (Fig. 1b). We also evaluated the correlations between the ICAM-1 promoter methylation level and clinical parameters. No correlations were found between the promoter methylation level and age or gender (*P* > 0.05).

### Detection of global DNA methylation pattern

**Fig. 3:**
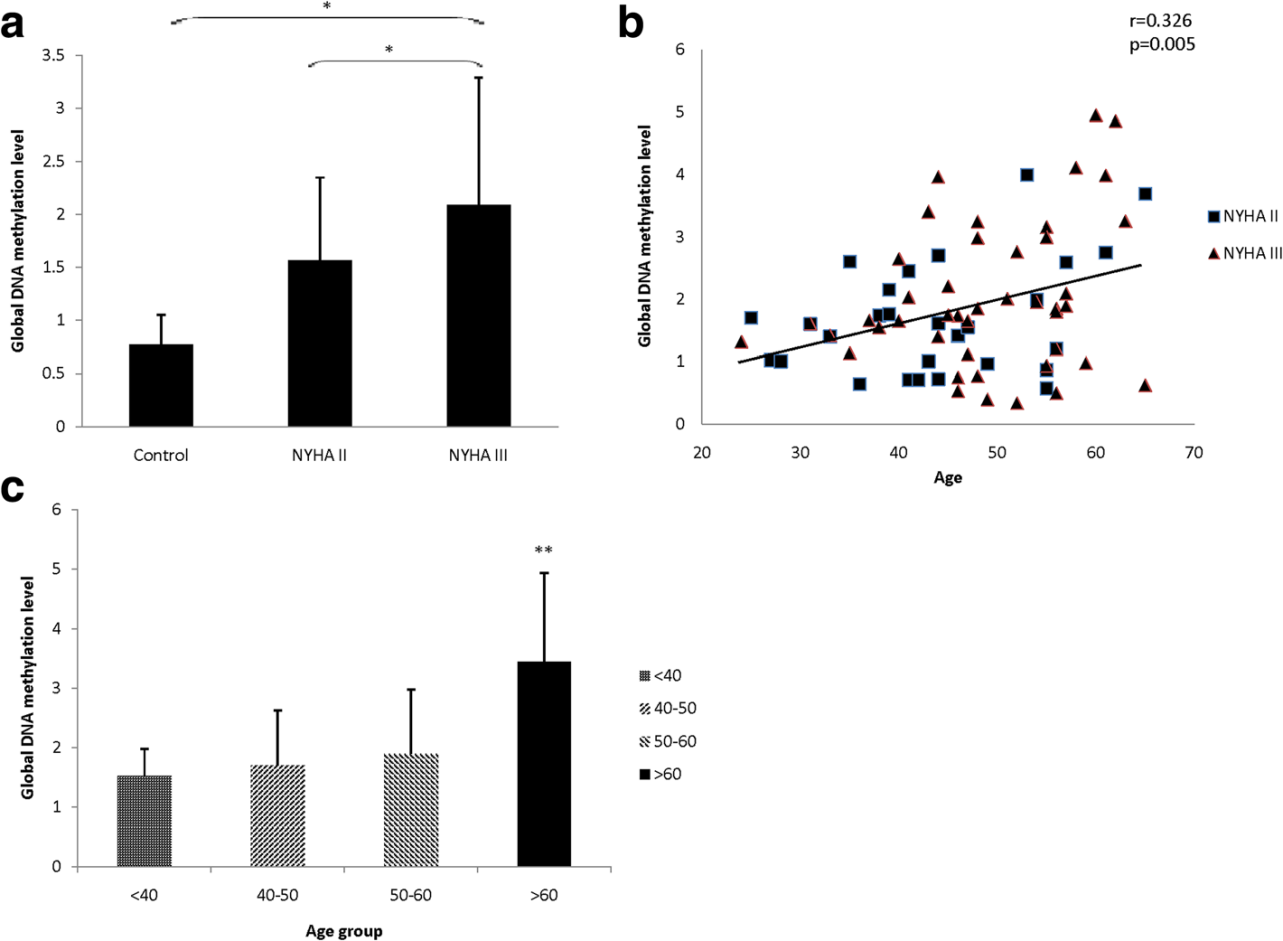
Global DNA methylation levels in different group and the correlation with age. **a** Shows the global DNA methylation levels of 3 group. Compared with healthy control and NYHA IIgroup, the global DNA methylation level is significantly higher in NYHA III group. **b** Shows the average global DNA hypermethylation status had positive correlation with age. **c** Shows the average global DNA methylation status was significantly higher in 60 years older group, compared with other three age groups.(^*^, *p* < 0.05; ^**^, *p* < 0.01)

The global DNA methylation levels were significantly increased in NYHA III group (healthy control vs. NHYA III, 0.77 ± 0.28 vs. 2.09 ± 1.20, *p* = 0.017; NYHA IIvs. NHYA III, 1.57 ± 0.78 vs. 2.09 ± 1.20, *p* = 0.040). No significant difference was found between healthy controls and NYHA II patients (*P* = 0.151) (Fig. 3a). Other associations were also found: the average global DNA methylation status had positive correlation with age (*r* = 0.326, *p* = 0.005) (Fig. 3b). For four age groups (<40 years, 40–50 years, 50–60 years and >60 years), the average global DNA methylation level was significantly increased in 60 years older group than other three age groups (1.52 ± 0.46, 1.70 ± 0.93, 1.90 ± 1.08 vs. 3.45 ± 1.48, *p* = 0.006) (Fig. 3c). No correlation was found between the global DNA methylation levels and gender (*P* > 0.05).

### Expression of DNMTs in right atrial myocardium tissue

**Fig. 4:**
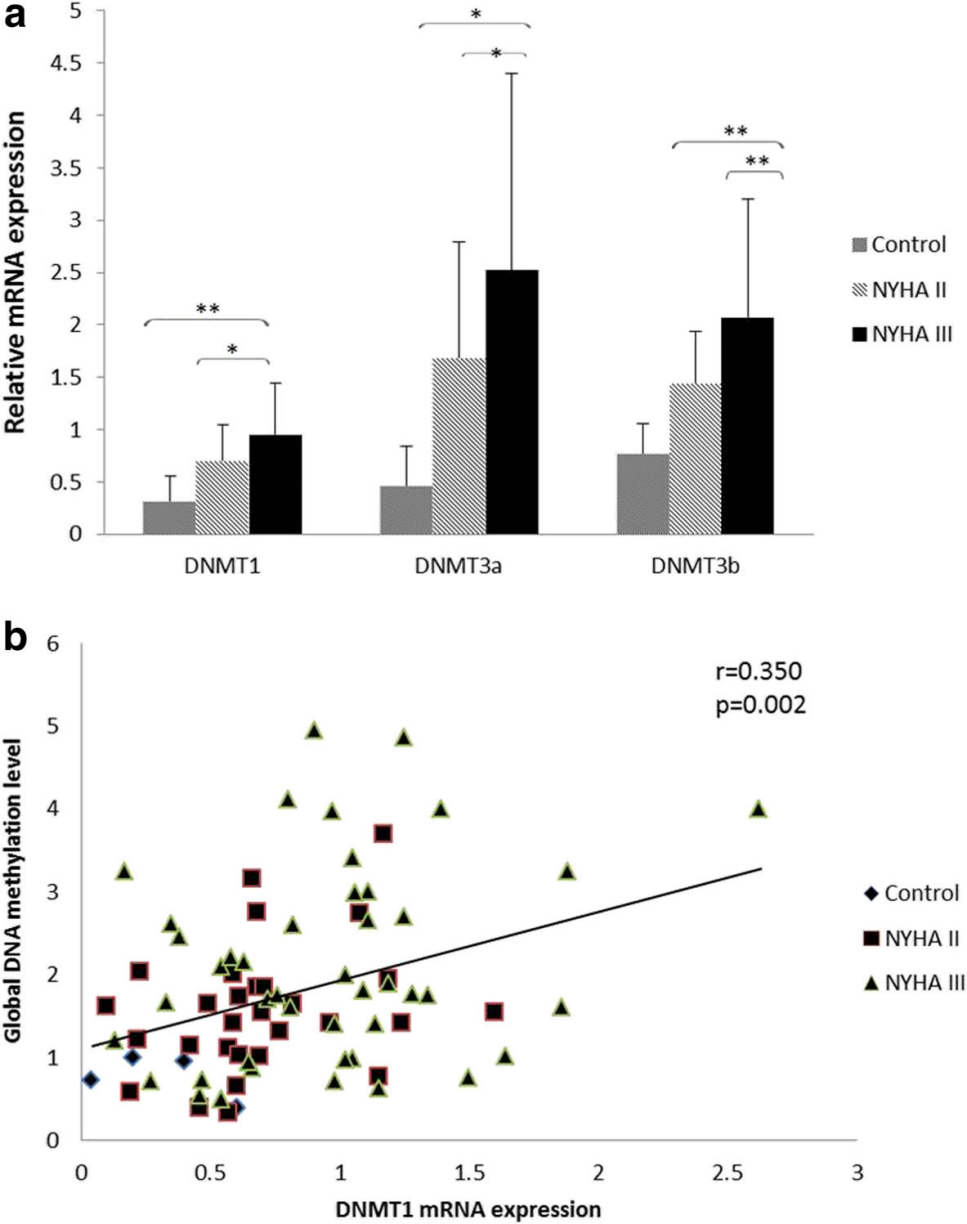
**a** Shows The expression levels of all 3 DNMTs genes were significantly upregulated in NYHA III group, compared with healthy control and NYHA IIgroup. (^*^, *p* < 0.05; ^**^, *p* < 0.01). **b** shows DNMT1 mRNA expression levels had significant positive correlation with the OD value of global methylation levels (*r* = 0.350, *p* = 0.002)

The mRNA expression levels of DNMTs were significantly increased in the NHYA III group. For DNMT1 (healthy control vs. NHYA III, 0.31 ± 0.24 vs. 0.95 ± 0.49, *p* = 0.006; NYHA IIvs. NHYA III, 0.70 ± 0.35 vs. 0.95 ± 0.49, *p* = 0.018). No significant difference was found between healthy controls and NYHA II patients (*P* = 0.094). For DNMT3a (healthy control vs. NHYA III, 0.46 ± 0.38 vs. 2.52 ± 1.88, *p* = 0.016; NYHA IIvs. NHYA III, 1.68 ± 1.11 vs. 2.52 ± 1.88, *p* = 0.030). No significant difference was found between healthy controls and NYHA II patients (*P* = 0.156). For DNMT3b (healthy control vs. NHYA III, 0.77 ± 0.29 vs. 2.07 ± 1.13, *p* = 0.008; NYHA IIvs. NHYA III, 1.44 ± 0.50 vs. 2.07 ± 1.13, *p* = 0.005). No significant difference was found between healthy controls and NYHA II patients (*P* = 0.169) (Fig. 4a).

Meanwhile, DNMT1 mRNA expression levels had significant positive correlation with the global DNA methylation levels (*r* = 0.350, *p* = 0.002) (Fig. 4b). No correlations were found between the global DNA methylation levels and DNMT3a or DNMT3b mRNA expression levels (*r* = 0.221, *p* = 0.053, for DNMT3a; *r* = 0.181, *p* = 0.115, for DNMT3b).

## Discussion

Evidence shows that the combinations of environmental and heritable agents are important initial factors for complex disorders. Epigenetic regulation, which was considered to be an important environmental factor of disease outcome, had been investigated in many researches. Epigenetic means heritable changes in gene expression which will not change the primary DNA sequence. As one of the important epigenetic modifications, DNA methylation relates to genomic imprinting, DNA elements silencing and chromatin structure regulating, which lead to gene expression control eventually. A well characterized functional effect of DNA methylation is gene promoter hypomethylation relates to increased gene expression, whereas hypermethylation leads to transcriptional silencing [9, 10].

ICAM-1 gene is an important mediator for the necrosis of cardiac muscle; increased ICAM-1 gene expression could lead to cardiac muscle cells death and promote the progression of cardiac dysfunction. Meanwhile, ICAM-1 gene is an independent predictor of complications in heart failure patients [21–23]. In our study, DNA hypomethylation was found in the ICAM-1 gene promoter and this hypomethylation correlated with increased mRNA expression level of ICAM-1 gene in the NYHA III group. These evidences demonstrated that DNA hypomethylation correlated with the progression of RHVD patients.

It is known that global DNA hypomethylation is a common feature in cancer [12, 20]. This hypomethylation become more remarkable in older age and correlated with pathogenesis and progression in cancer [24]. However, limited studies are reported in RHVD. In our study, we investigated whether global DNA methylation could induce changes in RHVD. We found the global DNA methylation levels are significantly increased in the NYHA III group patients than in the NYHA II group and controls. Our results are consistent with several reports related to global DNA methylation levels in cardiovascular diseases. Sharma et al. [25] showed that global DNA methylation levels were significantly increased in coronary artery disease patients. Kao et al. [26] also found that heart failure can induce cardiac hypermethylation. Watson et al. [27] proved that hypoxia-induced pro-fibrotic changes were correlated with global DNA hypermethylation. Zhang et al. [28] showed that global DNA methylation is related to inflammation in atherosclerosis. Evidences show that inflammation and auto-immune response contributes to the pathogenesis of RHVD [3, 4]. This means that global DNA hypermethylation related inflammation changes may have a tight relation with RHVD.

Aging is a key factor which is related to the global and promoter DNA methylation changes. Our results indicated that the global DNA methylation levels had positive correlation with age in RHVD patients. Especially in the higher age group (60 years older), the global DNA methylation levels were significantly higher than other 3 age-groups. This result correlated with the research performed by Sharma et al. [25]. The exact regulation mechanism is still not known. We just had the hypothesis that in RHVD patient, age-related global DNA hypomethylation is offset by gene promoter hypermethylation and result in global DNA methylation increased, especially in the higher age group.

The DNA methylation reaction is catalyzed and maintained by DNMTs [7]. DNMTs overexpression was observed in many diseases and use DNMTs antagonists could inhibit genes promoter methylation, which means that DNMTs may be used as targets for the treatment [7]. Then we detect the effect of DNMTs in the RHVD patients. These mainly have three DNMTs named DNMT1, DNMT3a and DNMT3b in humans [7]. DNMT1 ensures the pre-existing methylation patterns are faithfully copied; DNMT3a and DNMT3b are in charge of introducing new methylation [7]. Kao et al. [26] showed that DNMT1 increased and with Pitx2c promoter hypermethylation in heart failure atrium compared with the normal atrium. Tao et al. [29] had reported that DNMT3a plays an important role in RASSF1A mediated upregulation of ERK1/2 on cardiac fibrosis. In our study, all 3 DNMT genes were overexpression in the NYHA III group. Moreover, the expression of DNMT1 had positive correlation with global DNA hypermethylation, which means that the effect of DNMT1 is predominant and more important in the RHVD disease.

### Limitations

The study is a clinical research and has limitations inherent in samples come from clinical tissue. The normal control samples come from the transplanted cardiac patients. The quantity of patients and the family members’ consent would influence on selection. Relatively small normal healthy control sample sizes may affect the results.

## Conclusions

Our results suggest that DNA methylation might act as an important bridge to link epigenetic variation and RHVD progression. Increased DNA methylation, both global and ICAM-1 gene promoter levels, is related to cardiac fibrosis and inflammation and causes the progression of RHVD. Meanwhile, DNMT1 is an essential participant in this epigenetic regulation. All of these results provide potential insights into molecular mechanisms of RHVD with potential therapeutic implications.

## Abbreviations

DNMT: DNA methyltransferase
ICAM-1: Intercellular adhesion molecule-1
NYHA: New York heart association
RHVD: Rheumatic heart valve disease
RT-PCR: Real-time quantitative reverse transcriptase-polymerase chain reaction
